# Podocyte Foot Process Effacement Precedes Albuminuria and Glomerular Hypertrophy in CD2-Associated Protein Deficient Mice

**DOI:** 10.3389/fmed.2021.745319

**Published:** 2021-09-10

**Authors:** John M. Basgen, Jenny S. Wong, Justina Ray, Susanne B. Nicholas, Kirk N. Campbell

**Affiliations:** ^1^Department of Research, Stereology and Morphometry Laboratory, Charles R. Drew University of Medicine and Science, Los Angeles, CA, United States; ^2^Division of Nephrology, Icahn School of Medicine at Mount Sinai, New York, NY, United States; ^3^Division of Nephrology, David Geffen School of Medicine at University of California, Los Angeles, CA, United States

**Keywords:** CD2AP deficient mice, podocyte foot process effacement, albuminuria, kidney morphometry, glomerular volume, Cavalieri Principle, Delesse Principle

## Abstract

**Background:** Podocyte foot process effacement is a key histologic finding in proteinuric kidney disease. We previously showed that 3-week old CD2AP-deficient mice have significant proteinuria, glomerular hypertrophy and mesangial expansion. The goal of this study is to use morphometry to establish the temporal sequence of podocyte foot process effacement, glomerular volume expansion and albuminuria in *Cd2ap*^−/−^ mice by measuring these parameters at the 2-week time point.

**Methods:** Wild-type mice age 14 ± 1 days with the Cd2ap gene (WT, *N* = 5) and mice deficient for Cd2ap (*Cd2ap* KO, *N* = 5) were generated. Kidneys were harvested and fixed in 2.5% glutaraldehyde and processed for examination by light and electron microscopy. An average of 415.2 (range 268–716) grid points were counted for all the glomeruli, and quantification of glomerular volume from each kidney. Urine was collected the day prior to sacrifice for urine albumin-to-creatinine ratio (ACR) measurements.

**Results:** There was no difference in albuminuria [median (range) mg/g] between WT [212.2 (177.6–388.4) mg/g] vs. *Cd2ap* KO mice [203.3 (164.7–910.2) mg/g], *P* = 0.89; or glomerular volume 68,307[10,931] vs. 66,844[13,022] μm^3^, *p* = 0.92. The volume densities of glomerular components of the podocyte, capillary lumen and mesangium were not different for the two groups, *P* = 0.14, 0.14 and 0.17 respectively. However, foot process width was increased in *Cd2ap* KO 1128[286] vs. WT [374 ± 42] nm, *P* = 0.02.

**Conclusion:** Here we show that while 2-week old WT and *Cd2ap* KO mice have similar levels of albuminuria, glomerular and mesangial volume, *Cd2ap* KO mice have more extensive podocyte foot process effacement. The data suggests that podocyte injury is the initiating event leading to mesangial expansion and albuminuria in this model.

## Introduction

Kidney podocytes are terminally differentiated epithelial cells with a complex cellular morphology that form the final barrier to urinary protein loss (1). Podocyte foot process effacement on electron microscopy is a common feature of proteinuric kidney diseases. Numerous disease-causing genes encoding functional components of the podocyte actin cytoskeleton and slit diaphragm have been identified in human familial focal segmental glomerulosclerosis (FSGS) and nephrotic syndrome (2–4). Direct evidence that podocyte loss causes glomerulosclerosis has been provided by elegant animal models where a 20% loss in podocyte number results in mild persistent proteinuria and FSGS while a depletion of >40% results in high-grade proteinuria and decreased renal function (5, 6).

The temporal relationship between the onset of albuminuria and the development of podocyte foot process effacement is unclear and inconsistent. Albuminuria has been reported without foot process effacement in mouse models of type IV collagen alpha3 deletion, in rats treated with an anti-nephrin antibody and in a rat model of diabetes (7–9). In glomerular basement membrane laminin β2 deficient mice, albuminuria occurs seven days before foot process effacement (10). The opposite relationship has also been reported. For example, the classic descriptions of rat puromycin aminonucleoside nephropathy showed foot process effacement occurring a few days before proteinuria (11–13). In humans with type 1 diabetes mellitus albuminuria preceded effacement (14, 15). More recently, foot process effacement was found in six out of eight children with Fabry's disease without albuminuria or other renal abnormalities who underwent research biopsies prior to starting enzyme replacement therapy (16).

Accurate measurements of podocytes and podocyte effacement require appreciation of the 3-dimensional nature of the glomerulus and glomerular components that are typically observed as 2-dimensional structures on microscopic images. Studies using limited 2-dimensional profiles may mis-report glomerular data when expressing the number of podocyte profiles per glomerular profile area as a surrogate for density or number of glomeruli per kidney. Importantly, glomerular profile number is not directly related to the number of podocytes contained in the 3-dimensional glomerulus. For example, large glomeruli may have a greater probability of intersecting the section than small glomeruli, thus a bias toward overcounting large glomeruli compared to smaller glomeruli (17), and overestimating average glomerular volumes. To avoid the bias of large glomeruli having a greater probability of intersecting the section and thus being over-represented, disector sampling of pairs of sections can facilitate selection of the glomeruli to be measured (17). Importantly, the distance between the two sections must be less than the smallest glomerular diameter and only glomeruli that intersect the second section and not the first should be selected. Using electron microscopy, Farquhar, Vernier, and Good were the first to describe a “smearing” and loss of foot processes in children with proteinuria (18, 19). Powell was the first to quantitate these changes by counting the number of slit pores per length of GBM (20). More recently, researchers have measured individual foot process and calculated the average width of the foot processes (21–23). Direct measurement of foot process width has the problem of measuring the width of the foot processes as they curve around the capillary wall. Instead of measuring individual foot process widths we measured length of slit diaphragm per area of GBM (14, 15, 24). This can be accomplished by counting the number of slit diaphragm profiles and the number of intersections between grid lines and GBM resulting in the parameter length density of slit diaphragm per area of GBM. The reciprocal of this parameter is mean foot process width. It is less time consuming to count the number of slit diaphragm profiles and the number of intersections between the grid lines and the interface than to measure the individual foot process widths. This method also eliminates the problem of measuring the width of foot processes when they curve around non-straight GBM.

We previously identified a dendrin-dependent podocyte-mesangial crosstalk axis in mice lacking CD2-associated protein (*Cd2ap* KO) (25). At three weeks of age, the animals had heavy proteinuria, podocyte foot process effacement and significant mesangial and glomerular volume expansion (25), but the sequence of events was not examined. Here, we sought to establish the temporal sequence of albuminuria, podocyte foot process effacement and glomerular volume expansion in *Cd2ap* KO mice by measuring these parameters at the 2-week time point. We used design-based stereological methods to make measurements on 2-dimensional images to obtain structural information about the 3-dimensional glomeruli.

## Materials and Methods

### Animals

The study followed the Guide for the Care and Use of Laboratory Animals of the National Institutes of Health using a protocol approved by the Institutional Animals Care and Usage Committee of the Icahn School of Medicine at Mount Sinai. Global *Cd2ap* KO mice were obtained from Dr. Andrey Shaw (previously at Washington University, St. Louis, MO) (26). Mice with and without the Cd2ap gene were generated and genotype confirmed by PCR and five WT and four *Cd2ap* KO mice were analyzed. The day prior to sacrifice, urine was collected for determination of albumin-to-creatinine ratio (ACR). At age 14 ± 1 days, mice were anesthetized with isoflurane and injected with ketamine/xylazine. Kidneys were harvested, cut transversely, and approximately one third of each kidney was placed in 2.5% glutaraldehyde in PBS and shipped to the Morphometry and Stereology Laboratory at Charles R. Drew University of Medicine and Science for morphometric analysis.

### Albuminuria

Albuminuria was measured by enzyme-linked immunosorbent assay following the manufacturer's protocol (Bethyl Laboratories, Montgomery, TX). Creatinine was measured using the Creatinine Urinary Colorimetric Assay Kit (Cayman Chemicals, Ann Arbor, MI) following the manufacturer's protocol with the same urine samples for ACR.

### Kidney Morphometry

One-millimeter cubes were cut from the fixed kidney cortex, rinsed with buffer, post-fixed in 1% osmium tetroxide, and embedded in epoxy resin (Polybed 812, Polysciences, Warrington, PA) for light and electron microscopy analysis for quantification of the following parameters.

In order to examine glomerular components individually, the glomerulus was divided into four components: podocytes, mesangium including mesangial cells and mesangial matrix, capillary lumens including endothelial cells, and “other” including Bowman's space, GBM, and non-resolvable areas. The areal fraction of each component was measured using point counting (27). The volume fraction of each component is equal to the areal fraction if the measurements were done on random sections through the cortex-the Delesse Principle (28).

#### Glomerular Volume

Serial 1-μm thick sections were cut from the resin blocks using an EM UC7 ultramicrotome (Leica Microsystems, Buffalo Grove, IL) fitted with a Histo Jumbo diamond knife (Diatome US, Hatfield, PA). Every fifth section was saved to a microscope slide and stained with 1% toluidine blue. A total of 21 sections were saved from each kidney and the slides were labeled 0, 5, 10, 15, …, and 100. A BX51 microscope with a DP71 digital camera and DP Controller software (Olympus America, Cypress, CA) was used to observe the sections. A map of the glomerular profiles present in section 0 was made using the 10X objective lens. Glomeruli present in section 0 could not be used for volume measurement since an unknown volume of those glomeruli was lost. The map and subsequent sections were used to identify newly appearing glomeruli. Only glomeruli appearing and then disappearing within the stack of 21 sections could be used for analysis. The new glomeruli were mapped, numbered 1–7 (average 6.2, range 5–7 glomeruli per mouse) and imaged using the 100X objective lens. All profiles from each of the numbered glomeruli were imaged (average of 9.7, range 5–14 profiles per glomerulus).

**Figure 1:**
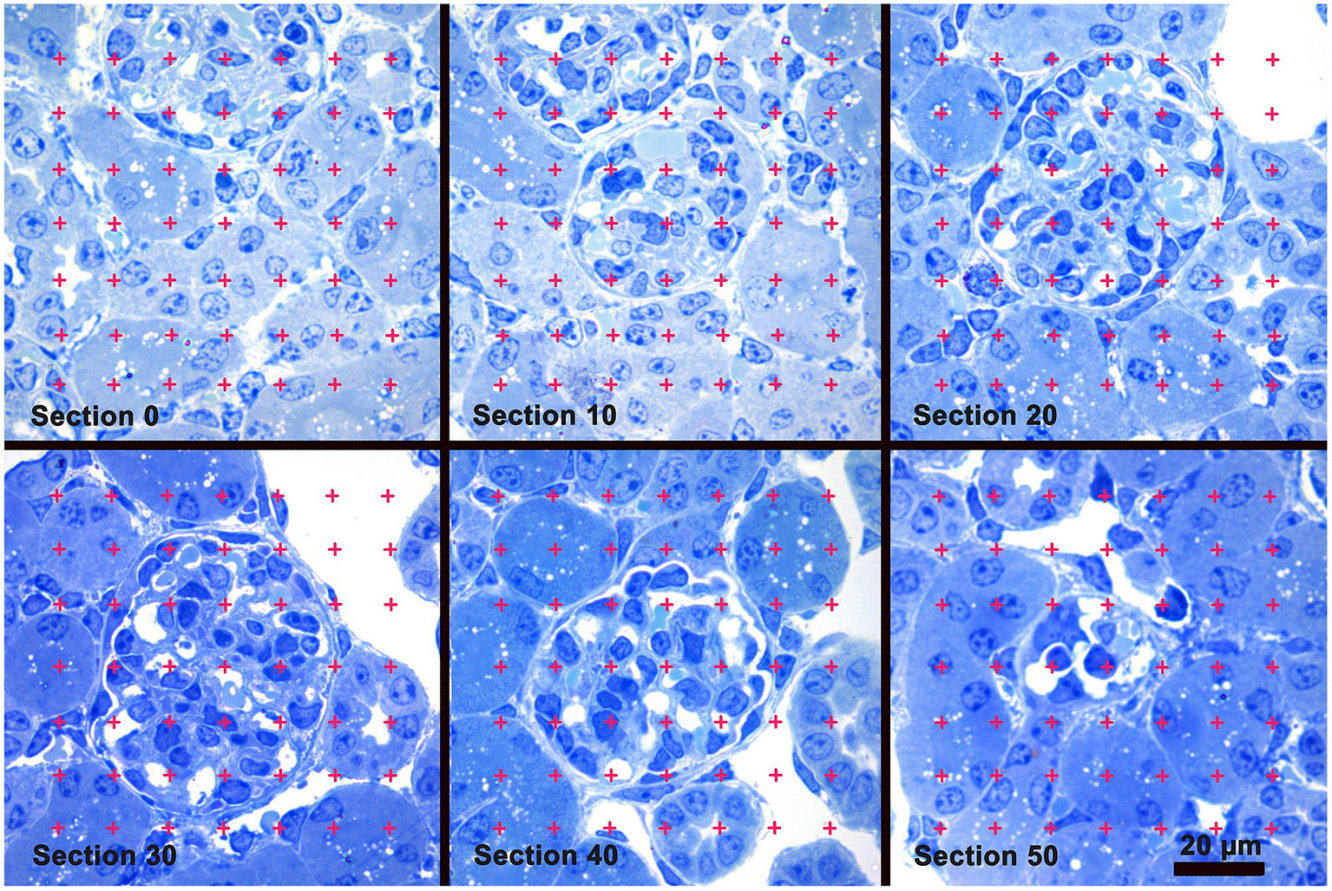
Glomerular Volume by the Cavalieri Principle. A sample of images from a stack of sections used to measure glomerular volume. Section 0 precedes the appearance of the glomerulus present in sections 10 thru 50. A counting grid was randomly placed over each glomerular profile and the number of grid points falling on the profiles was counted. Glomerular volume was calculated by multiplying the distance between the sections by the sum of the points falling on all the profiles from the glomerulus, and then multiplied by the area represented by one grid point. Toluidine blue stain.


An iMac computer with a 24′ monitor (Apple Inc., Cupertino, CA) and Photoshop software (Adobe Systems, San Jose, CA) were used to analyze the images. Using the Photoshop Polygonal Lasso tool a minimum string polygon was drawn around each glomerular tuft defining the limits of the glomerular profile. The Cavalieri Principle (27, 29) was used to determine glomerular volume by superimposing a grid of points over each profile from a glomerulus using the Layers function of Photoshop (Figure 1). The number of grid points falling on all of the profiles from a glomerulus was counted and the volume for each glomerulus was calculated using the equation:

$$\begin{array}{l} {\text{Volume}}_{\text{glomerulus}}=5\times \sum P_{\text{glomerulus}}\times {\left(25,000/1734\right)}^{2}\;\mu m^{3} \end{array}$$

where 5 was the distance in μm between the profiles of a glomerulus, ∑P_glomerulus_ was the sum of grid points falling on all the profiles from the glomerulus, 25,000 was the distance between points on the grid in μm, and 1734 was the magnification of the images. A stage micrometer was imaged to document the magnification. The mean glomerular volume for each mouse was determined by calculating the average of the individual glomerular volumes from the mouse.

#### Volume of Glomerular Components: Podocyte, Mesangium, Capillary Lumen, Other

Using the same ultramicrotome fitted with an Ultra diamond knife (Diatome US), a 1-μm thick scout section was cut from an epoxy block and used to identify complete glomerular profiles at least one large glomerular diameter from the edge of the block. Silver-grey sections were then cut, placed on formvar coated slot grids (1 × 2 mm slot) and stained with uranyl acetate and lead citrate. These sections were observed with a JEM 1200-EX electron microscope (JEOL USA, Inc., Peabody, MA) fitted with a digital camera and DigitalMicrographs software (Gatan, Inc., Pleasanton, CA). At an initial magnification of 2500X the complete glomerular profile was imaged. Small profiles needed a single image, while larger profiles needed up to four images that were fitted together using the Photoshop software to make a montage of the complete glomerular profile. An average of 5.0, range 4–6 glomerular profiles were analyzed per mouse.

**Figure 2:**
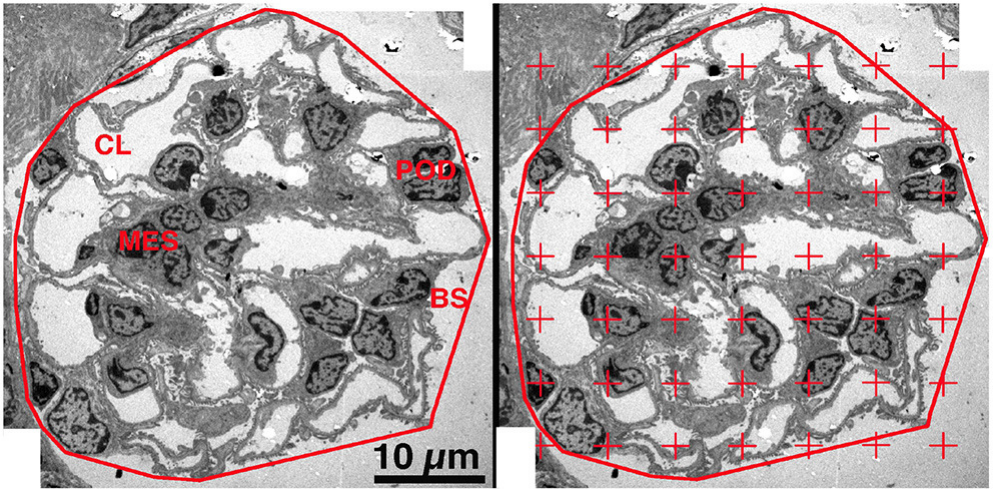
Areal density of glomerular components. **Left Panel**. On a low magnification EM image the glomerulus was defined by drawing a minimum string polygon around the tuft. Four components were defined: podocyte (POD), mesangium (MES), capillary lumen (CL), and remainder which included Bowman's space (BS) and GBM. **Right Panel**. A counting grid was randomly placed over the image and the number of points falling on each component is counted. The areal density of component X was calculated by dividing the number of grid points falling on component X by the total number of points falling on all four components.

Using the Polygonal Lasso tool of Photoshop, a minimum string polygon was drawn around the tuft defining the limits of the glomerular profile. The glomeruli were divided into four components: podocyte, mesangium, capillary lumen (including endothelial and circulating cells) and other (including Bowman's space and glomerular basement membrane) (Figure 2, Left Panel). To measure the areal density of each of the four components, a grid of points was superimposed over the glomerular profile using the Layers function of Photoshop and the number of points falling on each of the components was counted (28, 30) (Figure 2, Right Panel). The areal density of a component per glomerular profile was calculated using the equation:


$$\begin{array}{l} A_{\text{A }\left(\text{componentX}/\text{glomerulus}\right)}=\\ \sum P_{\text{componentX}}/\sum P_{\text{fourcomponents}}\;\mu m^{2}/\mu m^{2} \end{array}$$

where ∑P_componentX_ represents the sum of points falling on Component X, either podocyte, mesangium, capillary lumen or other for all the glomerular profiles from a kidney, and ∑P_fourcomponents_ is the sum of points falling on all the components from all glomerular profiles from a kidney. Because the areal densities were measured on random profiles from the glomeruli the measured areal densities equal the volume densities for each component according to the Delesse Principle (28):


$$\begin{array}{l} V_{\text{V }\left(\text{componentX}/\text{glomerulus}\right)=}\\ A_{\text{A }\left(\text{componentX}/\text{glomerulus}\right)\text{ μ}m^{3}/\mu m^{3}} \end{array}$$

The volume density does not determine the volumes of the components but only the ratio between the component volume and the glomerular volume. The individual component volumes were calculated using the equation:


$$\begin{array}{l} \text{Volum}{\text{e}}_{\text{componentX}}=\\ {\text{V}}_{\text{v}}\left(componentX/glomerulus\right)\;\times \;Volume_{\text{glomerulus}}\;\mu m^{3}. \end{array}$$

#### Podocyte Effacement

**Figure 3:**
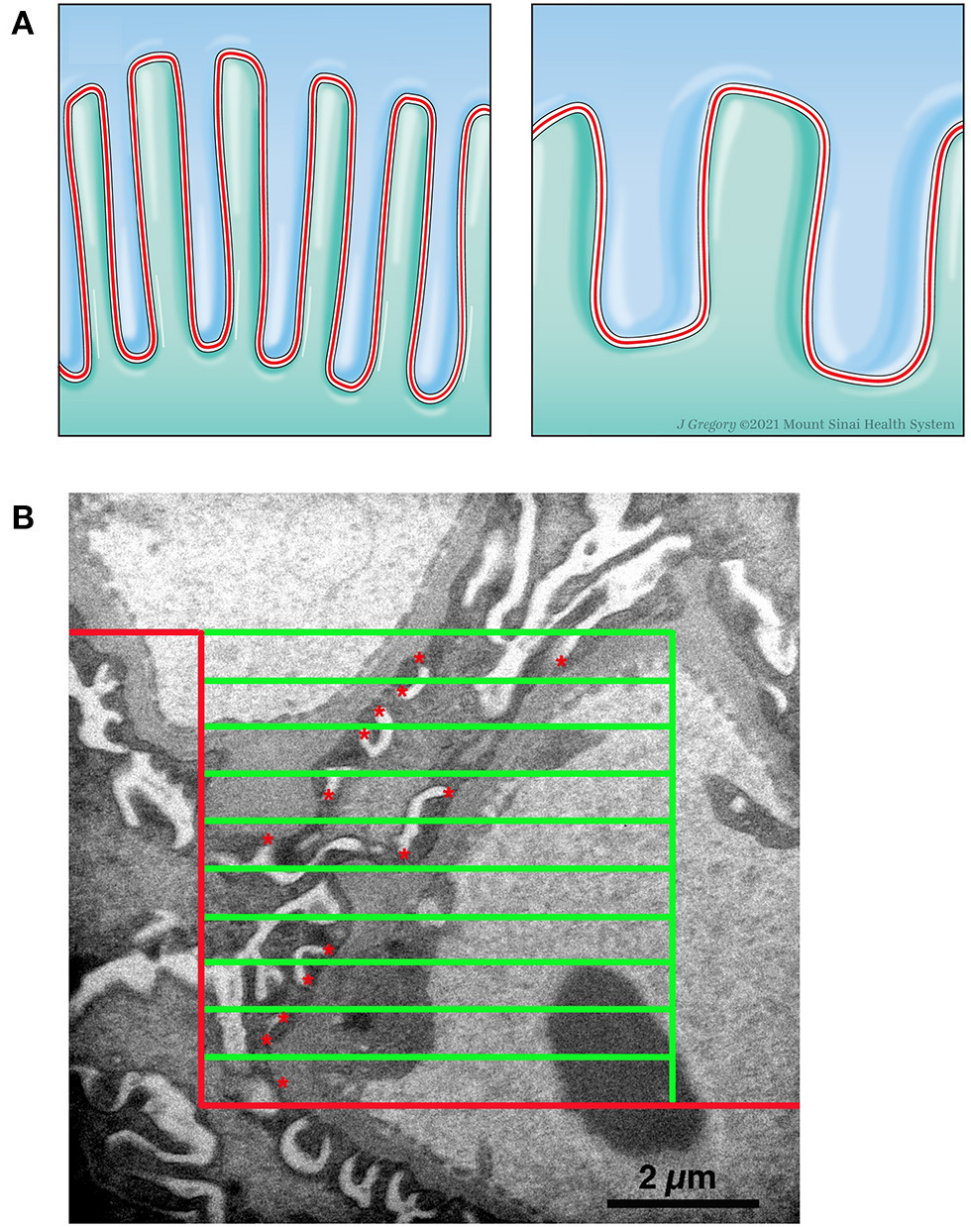
Measurement of length density of slit diaphragm. **(A) Left Panel**. Schematic drawing of two podocytes (green and blue) with normal interdigitating foot processes and the slit diaphragm (red line) between them. **Right Panel**. Schematic drawing of effacement with shortening of slit diaphragm. **(B)** Electron micrograph with line grid used to measure Ls. A counting frame was superimposed over a high magnification image. The frame consists of a peripheral guard zone, inclusion lines (vertical green line and upper horizontal green line), exclusion lines (red lines), and 10 counting lines (green horizontal lines). The number of times a counting line intersects the podocytes-GBM interface was counted and the number of slit diaphragm profiles (red *) within the counting frame and not touching the exclusion line was counted.


The conventional 2-dimensional picture of the normal structure of the podocyte in transmission electron microscopy images has foot processes “sitting” on GBM with a profile of a slit diaphragm located between adjacent foot processes. Actually, in the 3-dimensional glomerulus the slit diaphragm, a specialized cell-cell junction, meanders between the foot processes of adjacent podocytes and thus has a length that is beautifully demonstrated in the classical paper by Rodewald and Karnovsky (31) and illustrated in Figure 3A, Left Panel. The length of the slit diaphragm per area of the glomerular basement membrane (GBM) it sits on can be measured as the stereological parameter, length of slit diaphragm per GBM area [L_s_(slit/GBM)] (14, 32). The conventional characteristic of podocyte effacement is a widening of the foot processes. Another characteristic of podocyte effacement is a decrease of the L_s_(slit/GBM) (Figure 3A, Right Panel). We measured L_s_(slit/GBM) with the same glomeruli used to measure the volume of the glomerular components. A set of high magnification images was obtained systematically without bias from throughout each glomerular profile. An average of 36.0 (range 25–47) images per kidney were available for analysis. Using the Layers tool of Photoshop, a counting frame, consisting of inclusion, exclusion, and counting lines, and a guard zone surrounding the counting box was superimposed over each image. The guard zone eliminates ambiguous structures at the edge of the images. The number of intersections between the counting lines and the podocyte-GBM interface was counted as well as the number of slit diaphragm profiles within the counting frame and not touching the exclusion line (Figure 3B). The L_s_(Slit/GBM) was calculated using the equation:

$$\begin{array}{l} {\text{L}}_{\text{s}}\left(Slit/GBM\right)=\\ \sum Q_{\text{slit}}/\left(\sum I_{\text{Podo}-\text{GBM}}\times \left(22,500/37,500\right)\right)\;\mu m/\mu m^{2} \end{array}$$

where ∑Q_slit_ was the sum of slit diaphragm profiles counted on all the images from a kidney, ∑I_Podo−GBM_ was the sum of intercepts between the counting lines of the frame and the podocyte-GBM interface from all the images from a kidney, 22,500 was the distance in μm between frame lines, and 37,500 was the magnification of the images. A carbon replica was used to document the magnification of the images. The average foot process width is the reciprocal of the length density and was calculated using the equation:

$$\begin{array}{l} \text{Foot Process Width}=1/\left({\text{L}}_{\text{s}}\left(Slit/GBM\right)\;\times 1,000\right)\;nm. \end{array}$$

### Statistics

Morphometry data are presented as mean (SD). Group differences were compared using the Mann-Whitney U test. *P* <0.05 was set *a priori* as the level considered statistically significant.

## Results

### No Differences in Albuminuria Levels

**Figure 4:**
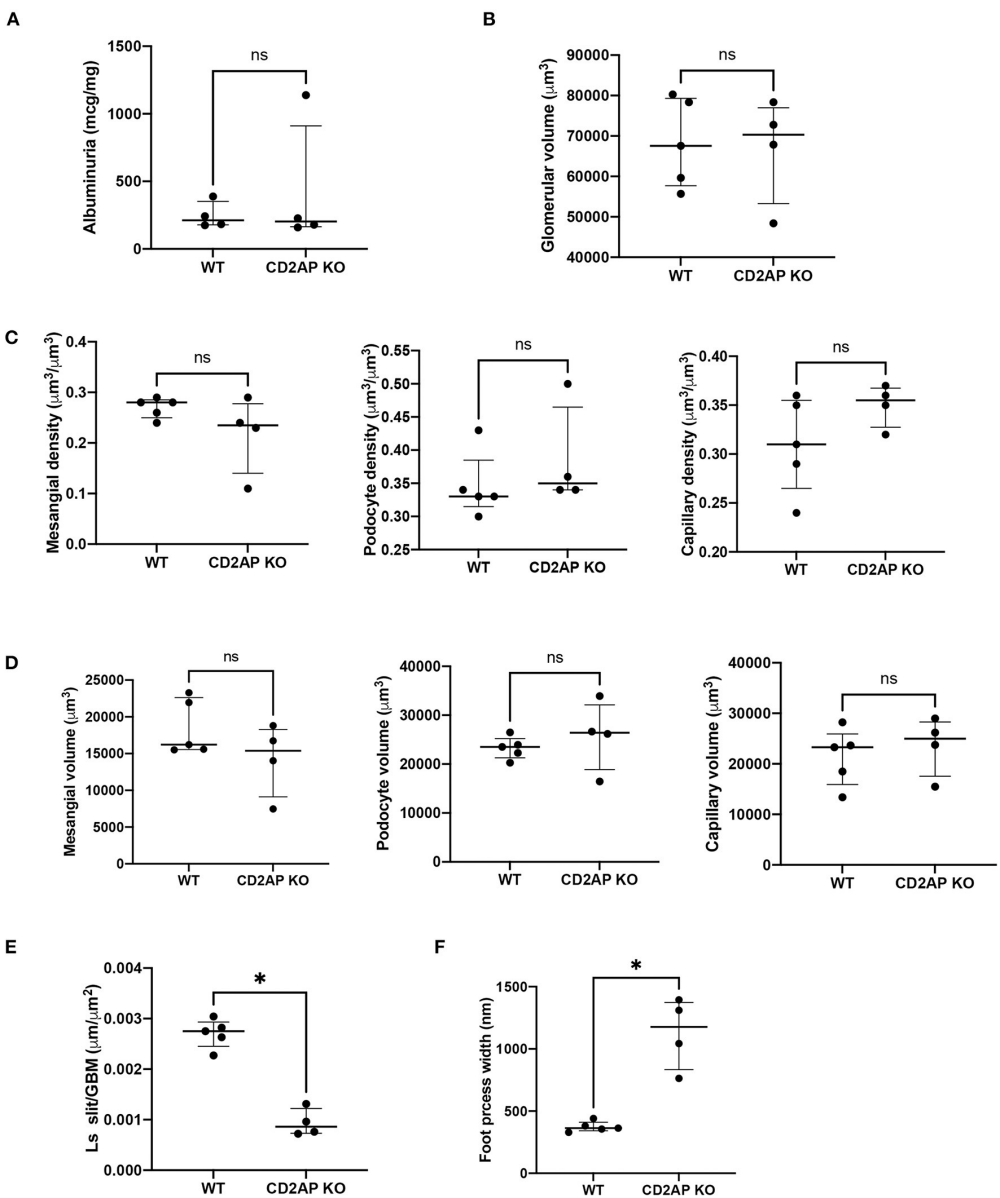
Albuminuria and morphometric data. **(A)** Plot of albuminuria showing no difference between WT and CD2AP KO groups. **(B)** Plot of glomerular volumes showing no difference between WT and CD2AP KO groups. **(C)** Plot of glomerular component volume densities showing no difference between WT and CD2AP KO groups for mesangium, podocyte or capillary lumen. **(D)** Plot of glomerular component volumes showing no difference between WT and CD2AP KO groups for mesangium, podocyte or capillary lumen. **(E)** Plot of length density per GBM area of the slit diaphragm showing a statistical difference between WT and CD2AP KO groups. **(F)** Plot of foot process width showing a statistical difference between WT and CD2AP KO groups. *p = 0.02.

Albuminuria levels were similar in WT vs. *Cd2ap* KO mice at 2 weeks of age. The median albumin/creatine ratio was 212.2 mg/g in WT mice [interquartile range (177.6–388.4) mg/g] vs. 203.3 mg/g in *Cd2ap* KO mice [164.7–910.2], *P* = 0.89 (Figure 4A).

### No Differences in Average Glomerular Volumes

At the time of sacrifice (14 ± 1days old) some glomeruli were not fully developed. To avoid measuring undeveloped podocytes only glomeruli at the capillary loop stage or older were used for analysis. Glomerular volume was measured by the Cavalieri Principle (25) using superimposed grid points. This method does not assume any particular shape of the glomeruli and measures the volume of individual glomeruli so that a distribution of glomerular volumes within a kidney can be obtained. A mean of 415.2 (range 268–716) grid points were counted for glomeruli from each kidney. The average glomerular volume was 68,307(10,931) μm^3^ for WT and 66,844(13,022) μm^3^ for *Cd2ap* KO mice, *P* = 0.92 (Figure 4B).

### No Differences in the Volume of Glomerular Components

We next measured the volume densities of the podocyte, capillary lumen, and mesangium components which were not different for the two groups, *P* = 0.17, 0.17, 0.27, respectively (Figure 4C). An average of 592 (range 387–1048) points for all the glomeruli/kidney were counted for determination of the volume densities of glomerular components. The volumes of the podocyte, capillary lumen, and mesangium components were calculated by multiplying the glomerular volume by the appropriate component volume density. There was no significant difference between the two groups, *P* = 0.41, 0.41, and 0.41 respectively (Figure 4D).

### Increased Podocyte Foot Process Width in Cd2ap^–/–^ Compared to Cd2ap^+/+^

L_s_ (slit/GBM) was determined on the same glomeruli used to measure the volume of the glomerular components. An average of 230 (range 65–382) intercepts was counted from all the glomeruli per kidney for measurement of the foot process effacement and an average of 260 (range 99–696) slit diaphragm profiles was counted from all the glomeruli per kidney. L_s_(Slit/GBM) was 0.0027 (0.0003) μm/μm^2^ for the WT and 0.0009 (0.0003) μm/μm^2^ for the *Cd2ap* KO mice. The difference between the two groups was statistically significant, P = 0.02 (Figure 4E). The values for foot process width were 374 (42) nm for WT mice and 1128 (286) nm for Cd2ap KO mice (Figure 4F). Since the foot process width is the reciprocal of the L_s_ (Slit/GBM) the *P*-value was the same.

## Discussion

This is the first study to report ACR and detailed kidney morphometric measurements for 2-week Cd2ap^+/+^ and Cd2ap^−/−^ mice. We showed similar results for ACR, volume of glomerular components but significantly increased podocyte foot process width in Cd2ap^−/−^ compared to Cd2ap^+/+^. The data indicates for the first time that podocyte injury is the initiating event that subsequently leads to mesangial volume expansion and glomerular changes in proteinuric disease progression.

The development of albuminuria has been linked to changes in several structures of the glomerulus including the components that comprise the filtration barrier (33). The podocyte plays an essential role in establishing the filtration barrier which consists of slits between interdigitating podocyte foot processes surrounding the glomerular capillaries of fenestrated endothelium and glomerular basement membrane (34, 35). Injury to podocytes, resulting in podocyte effacement has been observed in several experimental albuminuric/proteinuric models induced by toxins and/or genetic mutations (36), such as mutations in slit-diaphragm associated proteins, including CD2AP (26). Maintaining the integrity of the filtration barrier is a critical part in maintaining overall renal function, but whether podocyte effacement preceded the development of expansion of mesangial volume, which has been shown to correlate with progressive renal dysfunction (37) during loss of excess albumin had not previously been examined. Understanding the sequence of events has important implications, particularly in underlying disease pathology, but also for therapeutic strategies. Thus, the use of detailed morphometric analyses, as conducted in this study was necessary to identify the chronology of structural changes that might predict albuminuria. Indeed, it is possible some albumin that crosses the barrier is absorbed by the proximal tubules and therefore doesn't appear in the urine but Oken (38) and colleagues showed that only a small amount of albumin is absorbed by the tubules.

We previously published that an early morphometric abnormality in CD2AP KO mice was glomerular volume expansion due to increases in the mesangial compartment at 3 weeks of age (25). This was an interesting finding since CD2AP expression in glomeruli is limited to the podocyte. Indeed, in that study, a reduction in podocyte number did not occur until week 5. Our findings here confirm the importance of CD2AP expression in maintaining podocyte integrity with CD2AP loss causing significant foot process effacement. Taken together, these results suggest that the loss of CD2AP in podocytes causes podocyte injury (identified by foot process effacement on electron microscopy), drives pathogenic podocyte-mesangial crosstalk, ultimately leading to glomerular volume expansion, podocyte depletion and progressive glomerular disease progression. Albuminuria occurs after podocyte foot process effacement, but before podocyte depletion and is first significantly increased at the time of glomerular volume expansion.

This study has its limitations. First, we did not distinguish superficial from juxtamedullary glomeruli. There is some evidence that though variable, morphologic changes related ischemia and hyperfiltration may be more severe in juxtamedullary glomeruli (39). Second, while we performed detailed glomerular morphometric analyses in this study as well as in our previously published work with older CD2AP KO mice (25), here we took the additional step of measuring foot process width. This enabled us to achieve our stated goal of defining the temporal relationship between the development of albuminuria and the development of podocyte foot process effacement. Third, depending on developmental stage podocytes possess a diverse repertoire of intercellular junctional components including tight, adherens and gap junctions (40). The slit diaphragm is a modified adherens junction (41). Under nephrotic conditions, tight junction complexes have been reported to appear at foot process interfaces to replace the diminished or displaced slit diaphragm (42). Our cellular junction analysis was limited to slit diaphragm length density per GBM area and did not include other cell-cell adhesion measurements. Finally, the sample size of 4 animals per group is small and limited to one glomerular disease model. Further studies will be needed to establish generalizability given the heterogeneity of proteinuric kidney disease.

While CD2AP is a disease causing gene for human FSGS, the temporal relationship between albuminuria and morphometric glomerular changes has implications beyond the CD2AP KO experimental model and genetic causes of nephrotic syndrome. Albuminuria is typically the first clinical sign that a patient has glomerular disease. Our data suggests that this could be a delayed finding after significant podocyte actin cytoskeletal injury has already occurred. Podocyte foot process effacement is a dynamic process that involves a redistribution of two actin networks: central actin bundles and the cortical actin network beneath the plasma membrane (43). Simplification and retraction of individual foot process, impaired adhesion properties and ultimately, detachment can result. The findings therefore highlight the need for assays that can better detect subclinical podocyte injury. A good example of this was recently shown in a small study where 6 of 8 patients with classic Fabry disease, but normal kidney function and no albuminuria were found to have podocyte foot process effacement on kidney biopsy (16). Foot process widening has also long been described in normoalbuminuric patents with type I diabetes (44). Since podocytes are terminally differentiated with a limited capacity to regenerate (45), with a critical reduction in podocyte number of >40% considered the “point of no return” beyond which proteinuric kidney disease progresses in a committed manner (5), early identification of podocyte injury is essential.

## Data Availability Statement

The original contributions presented in the study are included in the article/supplementary material, further inquiries can be directed to the corresponding author/s.

## Ethics Statement

The animal study was reviewed and approved by Icahn School of Medicine at Mount Sinai IACUC Committee.

## Author Contributions

JB, SN, and KC: conceptualization and supervision. JB: kidney morphometry. JW and JR: animal data collection and ELISA. JB, JW, JR, KC, and SN: manuscript draft writing, review, and editing. All authors have read and agreed to the published version of the manuscript.

## Funding

This work was supported by NIH grant R01 DK103022 and the National Institute on Minority Health and Health Disparities (Grant # U54-MD007598).

## Conflict of Interest

The authors declare that the research was conducted in the absence of any commercial or financial relationships that could be construed as a potential conflict of interest.

## Publisher's Note

All claims expressed in this article are solely those of the authors and do not necessarily represent those of their affiliated organizations, or those of the publisher, the editors and the reviewers. Any product that may be evaluated in this article, or claim that may be made by its manufacturer, is not guaranteed or endorsed by the publisher.

## References

[1] Kriz W Lemley KV. The role of the podocyte in glomerulosclerosis. Curr Opin Nephrol Hypertens. (1999) 8:489–97. 10.1097/00041552-199907000-00014 10491745

[2] Woroniecki RP Kopp JB. Genetics of focal segmental glomerulosclerosis. Pediatr Nephrol. (2007) 22:638–44. 10.1007/s00467-007-0445-y 17347836 PMC2467504

[3] Antignac C. Molecular basis of steroid-resistant nephrotic syndrome. Nefrologia. (2005) 25(Suppl. 2):25–8. 16050398

[4] Pollak MR. The genetic basis of FSGS and steroid-resistant nephrosis. Semin Nephrol. (2003) 23:141–6. 10.1053/snep.2003.50014 12704574

[5] Wharram BL Goyal M Wiggins JE Sanden SK Hussain S Filipiak WE. Podocyte depletion causes glomerulosclerosis: diphtheria toxin-induced podocyte depletion in rats expressing human diphtheria toxin receptor transgene. J Am Soc Nephrol. (2005) 16:2941–52. 10.1681/ASN.2005010055 16107576

[6] Kim YH Goyal M Kurnit D Wharram B Wiggins J Holzman L. Podocyte depletion and glomerulosclerosis have a direct relationship in the PAN-treated rat. Kidney Int. (2001) 60:957–68. 10.1046/j.1523-1755.2001.060003957.x 11532090

[7] Kalluri R. Proteinuria with and without renal glomerular podocyte effacement. J Am Soc Nephrol. (2006) 17:2383–9. 10.1681/ASN.2006060628 16914535

[8] Topham PS Kawachi H Haydar SA Chugh S Addona TA Charron KB. Nephritogenic mAb 5-1-6 is directed at the extracellular domain of rat nephrin. J Clin Invest. (1999) 104:1559–66. 10.1172/JCI7728 10587519 PMC409863

[9] Steffes MW Leffert JD Basgen JM Brown DM Mauer SM. Epithelia cell foot process width in intact and uninephrectomized diabetic and nondiabetic rats. Lab Invest. (1980) 43:225–30. 6447233

[10] Jarad G Cunningham J Shaw AS Miner JH. Proteinuria precedes podocyte abnormalities inLamb2-/- mice, implicating the glomerular basement membrane as an albumin barrier. J Clin Invest. (2006) 116:2272–9. 10.1172/JCI28414 16886065 PMC1523402

[11] Harkin JC Recant L. Pathogenesis of experimental nephrosis electron microscopic observations. Am J Pathol. (1960) 36:303–29. 14399816 PMC1942209

[12] Ericsson JL Andres GA. Electron microscopic studies on the development of the glomerular lesions in aminonucleoside nephrosis. Am J Pathol. (1961) 39:643–63. 19971009 PMC1942421

[13] Nevins TE Gaston T Basgen JM. Quantitative indexes of aminonucleoside-induced nephrotic syndrome. Am J Pathol. (1984) 117:30–6. 6486243 PMC1900560

[14] Toyoda M Najafian B Kim Y Caramori ML Mauer M. Podocyte detachment and reduced glomerular capillary endothelial fenestration in human type 1 diabetic nephropathy. Diabetes. (2007) 56:2155–60. 10.2337/db07-0019 17536064

[15] Bjorn SF Bangstad HJ Hanssen KF Nyberg G Walker JD Viberti GC. Glomerular epithelial foot processes and filtration slits in IDDM patients. Diabetologia. (1995) 38:1197–204. 10.1007/s001250050412 8690172

[16] Tondel C Kanai T Larsen KK Ito S Politei JM Warnock DG. Foot process effacement is an early marker of nephropathy in young classic fabry patients without albuminuria. Nephron. (2015) 129:16–21. 10.1159/000369309 25531941

[17] Sterio DC. The unbiased estimation of number and sizes of arbitrary particles using the disector. J Microsc. (1984) 134:127–36. 10.1111/j.1365-2818.1984.tb02501.x 6737468

[18] Farquhar MG Vernier RL Good RA. Studies on familial nephrosis. II Glomerular changes observed with the electron microscope. Am J Pathol. (1957) 33:791–817. 13444463 PMC1934695

[19] Farquhar MG Vernier RL Good RA. An electron microscope study of the glomerulus in nephrosis, glomerulonephritis, and lupus erythematosus. J Exp Med. (1957) 106:649–60. 10.1084/jem.106.5.649 13475621 PMC2136823

[20] Powell HR. Relationship between proteinuria and epithelial cell changes in minimal lesion glomerulopathy. Nephron. (1976) 16:310–7. 10.1159/000180616 765875

[21] Gundersen HJ Seefeldt T Osterby R. Glomerular epithelial foot processes in normal man and rats. Distribution of true width and its intra- and inter-individual variation. Cell Tissue Res. (1980) 205:147–55. 10.1007/BF00234450 7363304

[22] Bohman SO Jaremko G Bohlin AB Berg U. Foot process fusion and glomerular filtration rate in minimal change nephrotic syndrome. Kidney Int. (1984) 25:696–700. 10.1038/ki.1984.76 6482174

[23] Ellis EN Steffes MW Chavers B Mauer SM. Observations of glomerular epithelial cell structure in patients with type I diabetes mellitus. Kidney Int. (1987) 32:736–41. 10.1038/ki.1987.268 3430959

[24] Najafian B Tondel C Svarstad E Gubler MC Oliveira JP Mauer M. Accumulation of globotriaosylceramide in podocytes in fabry nephropathy is associated with progressive podocyte loss. J Am Soc Nephrol. (2020) 31:865–75. 10.1681/ASN.2019050497 32127409 PMC7191924

[25] Weins A Wong JS Basgen JM Gupta R Daehn I Casagrande L. Dendrin ablation prolongs life span by delaying kidney failure. Am J Pathol. (2015) 185:2143–57. 10.1016/j.ajpath.2015.04.011 26073036 PMC4530132

[26] Shih NY Li J Karpitskii V Nguyen A Dustin ML Kanagawa O. Congenital nephrotic syndrome in mice lacking CD2-associated protein. Science. (1999) 286:312–5. 10.1126/science.286.5438.312 10514378

[27] Nyengaard JR. Stereologic methods and their application in kidney research. J Am Soc Nephrol. (1999) 10:1100–23. 10.1681/ASN.V1051100 10232698

[28] Weibel ER. Stereological Methods. London: Academic Press (1979).

[29] Bai XY Basgen JM. Podocyte number in the maturing rat kidney. Am J Nephrol. (2011) 33:91–6. 10.1159/000322701 21196721 PMC3030545

[30] Basgen JM Sobin C. Early chronic low-level lead exposure produces glomerular hypertrophy in young C57BL/6J mice. Toxicol Lett. (2014) 225:48–56. 10.1016/j.toxlet.2013.11.031 24300173 PMC3957482

[31] Rodewald R Karnovsky MJ. Porous substructure of the glomerular slit diaphragm in the rat and mouse. J Cell Biol. (1974) 60:423–33. 10.1083/jcb.60.2.423 4204974 PMC2109155

[32] Mayhew TM. Basic stereological relationships for quantitative microscopical anatomy—a simple systematic approach. J Anat. (1979) 129:95–105. 511775 PMC1233086

[33] Haraldsson B Nystrom J Deen WM. Properties of the glomerular barrier and mechanisms of proteinuria. Physiol Rev. (2008) 88:451–87. 10.1152/physrev.00055.2006 18391170

[34] Huber TB Benzing T. The slit diaphragm: a signaling platform to regulate podocyte function. Curr Opin Nephrol Hypertens. (2005) 14:211–6. 10.1097/01.mnh.0000165885.85803.a8 15821412

[35] Menon MC Chuang PY He CJ. The glomerular filtration barrier: components and crosstalk. Int J Nephrol. (2012) 2012:749010. 10.1155/2012/749010 22934182 PMC3426247

[36] Reiser J Altintas MM. Podocytes. F1000Res. (2016) 5:F1000. 10.12688/f1000research.7255.1 26918173 PMC4755401

[37] Dalla Vestra M Saller A Mauer M Fioretto P. Role of mesangial expansion in the pathogenesis of diabetic nephropathy. J Nephrol. (2001) 14(Suppl. 4):S51–7. 11798146

[38] Oken DE Cotes SC Mende CW. Micropuncture study of tubular transport of albumin in rats with aminonucleoside nephrosis. Kidney Int. (1972) 1:3–11. 10.1038/ki.1972.2 5075945

[39] Newbold KM Sandison A Howie AJ. Comparison of size of juxtamedullary and outer cortical glomeruli in normal adult kidney. Virchows Arch A Pathol Anat Histopathol. (1992) 420:127–9. 10.1007/BF02358803 1549901

[40] Grahammer F Schell C Huber TB. The podocyte slit diaphragm—from a thin grey line to a complex signalling hub. Nat Rev Nephrol. (2013) 9:587–98. 10.1038/nrneph.2013.169 23999399

[41] Reiser J Kriz W Kretzler M Mundel P. The glomerular slit diaphragm is a modified adherens junction. J Am Soc Nephrol. (2000) 11:1–8. 10.1681/ASN.V1111 10616834

[42] Kurihara H Anderson JM Kerjaschki D Farquhar MG. The altered glomerular filtration slits seen in puromycin aminonucleoside nephrosis and protamine sulfate-treated rats contain the tight junction protein ZO-1. Am J Pathol. (1992) 141:805–16. 1415478 PMC1886648

[43] Schell C Huber TB. The evolving complexity of the podocyte cytoskeleton. J Am Soc Nephrol. (2017) 28:3166–74. 10.1681/ASN.2017020143 28864466 PMC5661293

[44] Torbjornsdotter TB Perrin NE Jaremko GA Berg UB. Widening of foot processes in normoalbuminuric adolescents with type 1 diabetes. Pediatr Nephrol. (2005) 20:750–8. 10.1007/s00467-005-1829-5 15827743

[45] Greka A Mundel P. Cell biology and pathology of podocytes. Annu Rev Physiol. (2012) 74:299–323. 10.1146/annurev-physiol-020911-153238 22054238 PMC3600372

